# Premises for Cholecystokinin and Gastrin Peptides in Diabetes Therapy

**DOI:** 10.1177/1179551419883608

**Published:** 2019-12-12

**Authors:** Jens F Rehfeld

**Affiliations:** Department of Clinical Biochemistry, Rigshospitalet, University of Copenhagen, Copenhagen, Denmark

**Keywords:** Cholecystokinin (CCK), diabetes mellitus, gastrin, gastrointestinal endocrinology, peptide drugs

## Abstract

Gastrin and cholecystokinin (CCK) are classical gastrointestinal peptide hormones. Their biogenesis, structures, and intestinal secretory patterns are well-known with the striking feature that their receptor-bound ‘active sites’ are highly homologous and that this structure is conserved for more than 500 million years during evolution. Consequently, gastrin and CCK are agonists for the same receptor (the CCK_2_ receptor). But in addition, tyrosyl O-sulphated CCK are also bound to the specific CCK_1_ receptor. The receptors are widely expressed in the body, including pancreatic islet-cell membranes. Moreover, CCK and gastrin peptides are at various developmental stages and diseases expressed in pancreatic islets; also in human islets. Accordingly, bioactive gastrin and CCK peptides stimulate islet-cell growth as well as insulin and glucagon secretion. In view of their insulinotropic effects, gastrin and CCK peptides have come into focus as drug targets, either alone or in combination with other insulinotropic gut hormones or growth factors. So far, modified CCK and gastrin peptides are being examined as potential drugs for therapy of type 1 as well as type 2 diabetes mellitus.

## Introduction

Four different lines of biomedical research during the last 4 decades are coming together in this review. The *first* entails the recognition that the digestive tract is by far the largest endocrine organ in the body. The gut expresses at least 20 different hormone genes, of which some are homologous. And as the prohormonal translation products are often heavily processed by endoproteolysis and amino acid derivatizations in neuroendocrine cells, the gastrointestinal tract releases an order of hundred different bioactive peptides to blood during and after a meal (for reviews, see Rehfeld^1,2^).

The *second* line is the gut-islet-axis, according to which some gastrointestinal hormones – not least gastrin and cholecystokinin (CCK) – in addition to the gut are expressed within pancreatic islet cells. The expression may occur in specific endocrine cells, in the classical islet cells, or in intra-islet ganglia and neurones. Furthermore, the level of expression varies during ontogenesis, phylogenesis, and during disease.^3-15^

The *third* line is the receptor-line, which has led to the recognition that receptors for hormonal gut peptides are widely expressed in extra-intestinal cells and organs. Hence, gut hormones contribute substantially to metabolic and growth regulation of a wide array of extra-intestinal functions all over the body. One of these functions is the secretion of insulin and glucagon from pancreatic islet cells.^16-18^ The gut hormones that stimulate in islet-cell secretion and growth have been named incretins (for review, see Rehfeld^19^).

The *last* line is pharmacochemical and deals with derivatization of bioactive peptides to become useful drugs (‘peptide therapeutics’).^20,21^ Recently, interest has focused on gut hormones with incretin-activity where, in particular, glucagon-like peptide-1 (GLP-1)-derived drugs have been applied to the treatment of type 2 diabetes mellitus.^22-24^ There are, however, considerable amounts of evidence to suggest that also other gastrointestinal hormones may prove valuable in diabetes therapy. Among these are the homologous CCK and gastrin peptides, which will be discussed in the following.

## CCK and Gastrin Peptides

**Figure 1.**
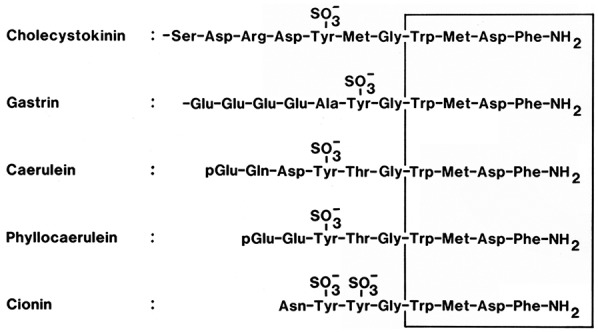
The C-terminal bioactive amino acid sequences of members of the gastrin/cholecystokinin family of peptides. Besides the sequences of mammalian cholecystokinin and gastrin, highly homologous sequences have been identified in extracts of frog skin glands (caerulein and phyllocaerulein) and the neural ganglion of the protochordate, *Ciona intestinalis* (cionin). Cionin with its disulphotyrosyl-containing sequence resembles a common ancestor candidate for gastrin and cholecystokinin.^25^

As shown in Figure 1, the C-terminal α-amidated ‘active site’ sequences of CCK and gastrin are highly homologous. A significant difference is the position of the C-terminal tyrosyl residue: position 7 in CCK vs position 6 in gastrin (as counted from the C-terminal Phe·NH_2_). However, not only is the sequence-position of the tyrosyl residue important but it is also noteworthy that in most of the CCK peptides, this residue is O-sulphated, whereas only half of the gastrins are tyrosyl O-sulphated.

The main production site of gastrin in adults is the antro-duodenal G-cells where most bioactive, ie, carboxyamidated, gastrin is synthesized as gastrin-17 and gastrin-34, both of which occur in tyrosyl O-sulphated and non-sulphated forms.^26-28^ Also, shorter (gastrin-14 and gastrin-6) as well as longer gastrins (gastrin-71) are synthesized and secreted, but only in small quantities.^29-31^ The synthesis of gastrin is cell-specific (for reviews, see Rehfeld,^1^ Rehfeld et al,^32^ and Schubert and Rehfeld^33^) Therefore, in the present context, it is noteworthy that the specific gastrin-producing cells in the foetal and neonatal pancreatic islets synthesize mainly O-sulphated gastrin-17.^4,34^ The sulphation, however, does not change the insulinotropic activity of gastrin. The expression of gastrin peptides also outside the gastrointestinal tract and the pancreas is summarized in Table 1.

**Table 1.** Expression of gastrin peptides in normal adult mammalian tissue.^a^

Tissue	Total translation product (pmol/g)	Precursor percentage
Gastrointestinal tract		
Antral mucosa	10 000	5
Duodenal mucosa	400	20
Jejunal mucosa	40	30
Ileal mucosa	20	85
Colonic mucosa	0.2	100
Neuroendocrine tissue		
Cerebellum	5	20
Vagal nerve	8	10
Adenohypophysis	200	98
Neurohypophysis	30	5
Adrenal medulla	2	100
Pancreas	2	95
Genital tract		
Ovaries	0.5	100
Testicles	6	100
Spermatozoa	2	55
Respiratory tract		
Bronchial mucosa	0.3	100

a Orders of magnitude based on examination of different mammalian species.

Like gastrin, CCK is also expressed in different molecular forms. The main forms are synthesized in endocrine I-cells in the duodenum, jejunum, ileum, and – in some species – also in the colon.^35,36^ The circulating forms released from the gut to plasma are CCK-58, CCK-33, CCK-22, and CCK-8.^32,37,38^ Notably, the predominant forms in blood are CCK-33 and CCK-58, whereas CCK-8 constitutes only a minor fraction in plasma.^37^ As already mentioned, most of the intestinal hormonal CCK peptides are O-sulphated, but around 25% are not.^39^ The CCK gene is, however, also abundantly expressed in cerebral and peripheral neurones, including pancreatic neurones that innervate islet cells and intrapancreatic ganglia.^8,40-43^ The major neurotransmitter forms are O-sulphated CCK-8 and the short CCK-5.^40,41,44,45^ CCK-5 and CCK-4 may be of particular interest in a diabetes context because of the high stimulatory potency for insulin release seen in the porcine and human pancreas.^8,46,47^ The tissue expression of CCK peptides also outside the intestinal tract is summarized in Table 2. CCK peptides in central and peripheral neurones are neurotransmitters, whereas the CCK in non-neuroendocrine cells is assumed to act as local, paracrine peptide messengers.

**Table 2.** Expression of CCK peptides in normal adult mammalian tissue.^a^

Tissue	Total translation product (pmol/g)	Precursor percentage
Gastrointestinal tract		
Duodenal mucosa	200	5
Jejunal mucosa	250	20
Ileal mucosa	20	50
Colonic mucosa	5	50
Neuroendocrine tissue		
Adenohypophysis	25	100
Neurohypophysis	20	10
Thyroid gland	2	20
Adrenal medulla	1	50
Genital tract		
Testicles	5	80
Spermatozoa^b^	–	–
Central nervous system		
Cerebral cortex	400	2
Hippocampus	350	2
Hypothalamus	200	2
Cerebellum	2	80

Abbreviation: CCK, cholecystokinin.

a Orders of magnitude based on examination of different mammalian species.

b Cholecystokinin peptides are present in spermatozoa of non-human mammals. The concentration, however, has not been quantitated.

## Gastrin and CCK Receptors

The targets for gastrin and CCK are 2 related G-protein coupled receptors.^48,49^ The original naming as CCK and gastrin receptor is simple and meaningful.^48,49^ But a later nomenclature with names such as CCK-A or CCK_1_ and CCK-B or CCK_2_ receptors, respectively, has now gained a strong foothold (for reviews, see Dufresne et al^18^ and Reubi^50^). Therefore, the CCK_1_/CCK_2_ receptor naming is used in the following.

The CCK_1_ receptor mediates gallbladder contraction, relaxation of the sphincter of Oddi, pancreatic growth and enzyme secretion, delay of gastric emptying, and inhibition of gastric acid secretion via somatostatin.^51^ The CCK_1_ receptor is also expressed in the pituitary, the myenteric plexus, and areas of the midbrain.^52,53^ The CCK_1_ receptor binds with high affinity only CCK peptides that are both carboxyamidated and tyrosyl O-sulphated, whereas the affinity of non-sulphated CCK peptides and gastrins is negligible.^54^ Thus, non-sulphated, longer CCKs, short CCKs (CCK-5 and CCK-4), and the gastrins – irrespective of their degree of sulphation – are not physiological agonists for the CCK_1_ receptor.

The CCK_2_ receptor is the predominant receptor for gastrin and CCK peptides in the central nervous system (‘the brain receptor’).^54,55^ It binds both sulphated and non-sulphated gastrin and CCK peptides, as well as short C-terminal fragments like CCK-5 and CCK-4 with high affinity. The CCK_2_ receptor is also abundantly expressed on enterochromaffin (ECL) cells in the stomach,^56,57^ and on islet cells and ganglionic neurones in the pancreas of man and pig.^16,58,59^ Thus, islet cells are targets for both locally released gastrin (from specific pancreatic gastrin-cells and β-cells),^3-7^ and CCK peptides (from intrapancreatic CCK neurones and islet cells),^8,10,11^ as well as from endocrine gastrin and CCK in circulation. Here, the concentrations of gastrin, however, are 10- to 20-fold above those of CCK.^37,38,60^ Notably, the CCK receptor expression in the pancreas is species-specific. There are major discrepancies between – on one hand – man and pig (abundant islet-cell expression of the CCK_2_ receptor) and – on the other hand – between rodents and dogs, where the specific CCK_1_ receptor is more abundant.^16,61,62^ Consequently, results on the insulinotropic effects of CCK and gastrin obtained from rat, mice, and dog studies do not necessarily apply to human physiology and diabetes pathophysiology.

## The Biological Linkage of Gastrin and CCK to Pancreatic Islets

As indicated above, an association between gastrin/CCK peptides and islet-cell functions (and hence a role for these peptides in diabetes therapy) has been discussed and examined in the last decades. The association includes a number of cellular, developmental, and pathological observations. First, the discovery that an essential part of gastrin in foetal and neonatal life in mammals is expressed in specific gastrin cells in pancreatic islets.^3-5^ These cells are ultra-structurally similar to the antro-duodenal G-cells in adults, but differ as being ‘closed’ cells without luminal contact and because the foetal pancreatic gastrin product is more extensively O-sulphated.^3-5^ The marked pancreatic expression precedes antral gastrin expression in the stomach,^3-5^ but low-level pancreatic expression is maintained also in adult life, although in inactive prohormonal forms.^6^ Second, the finding that pancreatic CCK neurones innervate endocrine islet cells and intra-islet ganglions involves also small CCK peptides in islet-cell regulation.^8,43^ Third, the observation that CCK_2_ receptors are expressed fairly abundantly on beta and alpha cells in human islets indicates that both gastrin and CCK peptides influence insulin and glucagon secretion.^16,59^ Fourth, there are also gastrin and CCK peptides in secretory granules within insulin cells of obese rodents and humans, where they apparently protect against β-cell apoptosis.^7,10,11,63,64^ Fifth, earlier literature has described islet-cell neogenesis and increased insulin secretion in endogenous hypergastrinaemia and during gastrin stimulation, emphasizing the growth stimulatory effects of gastrin and CCK peptides.^65-77^ Sixth, there is the well-known occurrence of gastrin- and CCK-producing neuroendocrine tumours from pancreatic islets.^78-83^ And, finally, there is the incretin effect of gastrin and CCK peptides as described below. The biological linkages of CCK and gastrin to islet cells are summarized in Table 3.

**Table 3.** Biological linkages of gastrin and/or CCK to pancreatic islets.

Peptide(s)	Linkage	References
Gastrin	Expression in islet G-cells during foeto-/neonatal life	3-7
CCK	Intra-islet CCK neurones	8, 42
Gastrin and CCK	CCK_2_ receptor expression on islet cells	16, 17, 58, 60, 61
CCK	Expression in β-cells during obesity	10, 11, 62
Gastrin	Islet-cell neogenesis and increase in the insulin secretion in hypergastrinaemia	65, 67, 69, 71-74
Gastrin and CCK	Neuroendocrine pancreatic tumours	77-82
Gastrin and CCK	Incretin effect, also during meals	64, 84
Gastrin and CCK	Potentiation of other incretins	74, 85, 86
Gastrin	Expression preceding islet-cell neogenesis after pancreatic duct-ligation	75

Abbreviation: CCK, cholecystokinin.

## Incretin Studies of Gastrin and CCK in Man and Pig

During the late 1960s and in the 1970s, a number of incretin studies of gastrin in man were reported from several laboratories.^46,47,65-67,69,84^ The conclusions in the 1970s from dose-response studies were that – on one hand – exogenous gastrin does indeed release insulin, but then – on the other hand – endogenous gastrin release after oral glucose in normal subjects was too small to explain the intestinal part of the insulin response during an oral glucose tolerance test.^65,71^ Therefore, using the oral-glucose-incretin definition, gastrin as such was assumed to contribute only little to the incretin effect of gastrointestinal hormones. However, review of the older studies suggests that this negative conclusion was false. Exogenous gastrin-17 in itself is a quite potent insulin-releaser together with intravenous glucose.^65^ Moreover, an ordinary protein-rich meal releases both gastrin and insulin in substantial amounts, whereas the elevation in blood glucose concentration is small.^65^ Hence, during and after such a meal, gastrin is likely to stimulate the secretion of insulin significantly. Moreover, studies in endogenous hypergastrinaemia in man support the idea of an incretin effect of gastrin in man.^66^

The incretin effect of CCK has been less extensively studied in man and pig; maybe because CCK studies entail several problems in comparison with those of gastrin. Thus, for exogenous studies, sufficient amounts of pure CCK peptides (especially CCK-58 and CCK-33) have been difficult to obtain. Moreover, larger CCKs are less stable than the gastrins, and the studies have been hard to monitor because of shortage of reliable CCK assays for plasma measurements of CCK.^38,60^ Nevertheless, short CCK peptides such as CCK-8, CCK-5, and CCK-4 have been shown to release insulin quite efficiently in man and in the isolated perfused porcine pancreas.^8,46,47,84,87^

## Gastrin and CCK Analogues for Diabetes Therapy

CCK and gastrin analogues for stimulation of insulin secretion will have to target the CCK_2_ receptor on the β-cells. Although CCK and gastrin peptides are all agonists for the CCK_2_ receptor (because of the common C-terminus Gly-Trp-Met-Asp-Phe·NH_2_ [see also Figure 1]), O-sulphated CCK peptides also activate the CCK_1_ receptor on the gallbladder. Receptor-activated permanent gallbladder contraction, however, is inexpedient and may result in cholelithiasis and other gallbladder problems.^82^ Therefore, sulphated CCK-like peptides should probably not be used for therapy of human diabetes.

The CCK analogues under study were recently reviewed.^88^ They include N-terminally glycosylated CCK-8 and other N-terminally protected CCK analogues (pGlu-Gln-CCK-8, and Ac-Y*-CCK-8).^85,86,89,90^ Of these, the pGlu-Gln-CCK-8 designed and tested by Irwin et al^90^ looks particularly promising, not least in combination with GLP-1.^86,91^

Also, gastrin alone, or in combination with GLP-1 or relevant growth factors, shows promise in treatments of type 1 diabetic rodents.^72-75,92^ Again, strikingly positive results were seen in the combinatorial treatment with gastrin and GLP-1.^75^ Accordingly, a hybrid dual agonist between GLP-1 and the C-terminal hexapeptide amide fragment of gastrin has proved pretty beneficial in diabetic mice.^93,94^

Interestingly, it has just been demonstrated that human β-cells after fibroblast growth factor 2 (FGF-2)-induced dedifferentiation express gastrin.^95^ And also worth mentioning is the fact that postprandial CCK-secretion is increased in Roux-en-Y gastric bypass (RYGB)-operated obese patients.^96-98^ Hence, endogenous CCK in these patients may contribute to the insulinotropic amelioration of their type 2 diabetes.

## Conclusions

Food is a prerequisite for life. Therefore, regulation of digestion is essential for all multicellular organisms. Accordingly, the gut is densely innervated and equipped with endocrine cells for accurate regulation of digestion, absorption and metabolic functions in the body. For decades, studies of gastrointestinal hormones have probably focused too much on functions inside the gut. Studies of incretin did for many years so to speak fall between 2 stools: the traditional gastrointestinal physiologists were more interested in proper gut functions (secretion of digestive juices, digestive enzymes, motility, and emptying), and classical endocrinologists did not like the darkness of the bowel.

With the rapidly growing epidemics of obesity and diabetes mellitus, incretin, however, has become a central biomedical issue. The prospect of GLP-1 analogues as major drugs for treatment of type 2 and perhaps also type 1 diabetes bears witness to this development and indicates that diabetes and obesity can be profoundly influenced by gastrointestinal hormones. Among these, GLP-1 and GIP (gastric inhibitory polypeptide or glucose-dependent insulinotropic polypeptide) are important, but not the only players. As described here and previously, the combinatorial effects of GLP-1 and GIP with CCK and gastrin peptides seem worth pursuing.^88,99-101^
